# Reporting, Monitoring, and Handling of Adverse Drug Reactions in Australia: Scoping Review

**DOI:** 10.2196/40080

**Published:** 2023-01-16

**Authors:** Joel Fossouo Tagne, Reginald Amin Yakob, Thu Ha Dang, Rachael Mcdonald, Nilmini Wickramasinghe

**Affiliations:** 1 Department of Health and Biostatistics, School of Health Sciences Swinburne University of Technology Melbourne Australia; 2 Centre for Health Analytics Murdoch Children's Research Institute Melbourne Australia; 3 MedTechVic Swinburne University of Technology Melbourne Australia; 4 Australian Association of Consultant Pharmacy Sydney Australia; 5 Department of Psychology, School of Health Sciences Swinburne University of Technology Melbourne Australia; 6 Behavioural Sciences Unit, Department of Health Services Research Peter MacCallum Cancer Centre Melbourne Australia; 7 Department of Nursing and Allied Health, Occupational Therapy Swinburne University of Technology Melbourne Australia; 8 Iverson Health Innovation Research Institute Swinburne University of Technology Melbourne Australia; 9 Epworth Healthcare Melbourne Australia

**Keywords:** pharmacovigilance, adverse drug reactions, primary care, digital health

## Abstract

**Background:**

Adverse drug reactions (ADRs) are unintended consequences of medication use and may result in hospitalizations or deaths. Timely reporting of ADRs to regulators is essential for drug monitoring, research, and maintaining patient safety, but it has not been standardized in Australia.

**Objective:**

We sought to explore the ways that ADRs are monitored or reported in Australia. We reviewed how consumers and health care professionals participate in ADR monitoring and reporting.

**Methods:**

The Arksey and O’Malley framework provided a methodology to sort the data according to key themes and issues. Web of Science, Scopus, Embase, PubMed, CINAHL, and Computer & Applied Sciences Complete databases were used to extract articles published from 2010 to 2021. Two reviewers screened the papers for eligibility, extracted key data, and provided descriptive analysis of the data.

**Results:**

Seven articles met the inclusion criteria. The Adverse Medicine Events Line (telephone reporting service) was introduced in 2003 to support consumer reporting of ADRs; however, only 10.4% of consumers were aware of ADR reporting schemes. Consumers who experience side effects were more likely to report ADRs to their doctors or pharmacists than to the drug manufacturer. The documentation of ADR reports in hospital electronic health records showed that nurses and pharmacists were significantly less likely than doctors to omit the description of the drug reaction, and pharmacists were significantly more likely to enter the correct classification of the drug reaction than doctors. Review and analysis of all ADR reports submitted to the Therapeutic Goods Administration highlighted a decline in physician contribution from 28% of ADR reporting in 2003 to 4% in 2016; however, within this same time period, hospital and community pharmacists were a major source of ADR reporting (ie, 16%). In 2014, there was an increase in ADR reporting by community pharmacists following the introduction of the GuildLink ADR web-based reporting system; however, a year later, the reporting levels dropped. In 2018, the Therapeutic Goods Administration introduced a black triangle scheme on the packaging of newly approved medicines, to remind and encourage ADR reporting on new medicines, but this was only marginally successful at increasing the quantity of ADR reports.

**Conclusions:**

Despite the existence of national and international guidelines for ADR reporting and management, there is substantial interinstitutional variability in the standards of ADR reporting among individual health care facilities. There is room for increased ADR reporting rates among consumers and health care professionals. A thorough assessment of the barriers and enablers to ADR reporting at the primary health care institutional levels is essential. Interventions to increase ADR reporting, for example, the black triangle scheme (alert or awareness) or GuildLink (digital health), have only had marginal effects and may benefit from further improvement revisions and awareness programs.

## Introduction

The World Health Organization defines pharmacovigilance (PV) as “the science and activities related to the detection, assessment, understanding and prevention of adverse effects of medications” [1]. Many harmful adverse drug reactions (ADRs) resulting from medication use go undetected or unreported to regulatory authorities [2]. The underreporting of ADRs remains a major threat to patient safety and is a substantial burden to established health care systems [2,3]. Most PV schemes are based on spontaneous and voluntary reporting [4]. In Australia, the Therapeutic Goods Administration (TGA) receive suspected ADRs reports from health care professionals (HCPs; physicians, pharmacists, nurses, etc) and the general public (consumers, patients, carers, members of the legal system, etc) [5,6]. The TGA regularly reviews available information originating from submitted ADR reports, and this informs drug safety decisions [4]. International studies have stated that less than 5% of ADRs are reported, including in jurisdictions where ADR reporting is mandatory [7]. Without a robust PV systems, ADRs may remain undetected for years, exposing patients to unanticipated health risks, and is a detriment to the health care system and taxpayers [8]. For example, the anti-inflammatory drug rofecoxib (Vioxx) was withdrawn from the market for high risk of myocardial infarctions after population exposures had reached millions, emphasizing the need for early detection of drug safety signals to ensure global health safety[7].

In Australia, approximately 400,000 consumers present to hospital emergency departments each year with medication-related problems [3,9]. Furthermore, about 7.2% to 11% of hospital admissions are specifically related to ADRs, of which approximately 50% are preventable [9,10]. Globally, it has been reported that 3.6% to 15.6% of hospital admissions are related to ADRs [11,12]. Complications related to ADRs may increase the mean length of hospital stay from 8 to 20 days [13]. According to the Pharmaceutical Society of Australia’s medication safety report (2019), health care expenditure for medication-related problems is estimated at AUD $1.4 billion (US $900,207) per annum [14]. Despite its limitations, spontaneous reporting remains the most common method for generating safety signals; however, it is estimated that only 6% of all ADRs that occur are reported [7,15,16]. The reasons for ADR underreporting have previously been described [10,17-19]; these may include limited knowledge and awareness of PV, reluctance to report by consumers or HCPs due to time constraints, and nonsupportive workplace structures. Although various interventions to improve ADR reporting have been implemented [20], ADR underreporting remains a limitation of the current PV system [10,21].

Previous studies have evaluated the effects of interventions for improving ADR reporting rates; however, the perspective of ADR reporting and handling within the Australian health care system remains largely unexplored [20-22]. There remains little integrative and collective knowledge on ADR reporting, monitoring, and handling in the Australian health care context [21]. This scoping review aimed to provide a comprehensive landscape of PV and ADR reporting, monitoring, and handling in Australia. This review may stimulate further research, policy makers, regulatory authorities, or software vendors to make decisions that may promote ADR reporting and improve patient safety within the Australian context.

## Methods

### Overview

The Arksey and O’Malley methodology framework was adopted for conducting this scoping review [23,24]. This framework uses a rigorous process of transparency that enables the replication of the search strategy, which increases the reliability of the study findings. The framework has six stages as follows.

### Stage 1: Identifying the Research Question

The focus of this scoping review was to explore ADR reporting in the Australian health care system and the extent of participation by consumers or HCPs and to describe the different reporting systems. The core questions are (1) How are ADRs monitored, reported, and handled in the Australian health care system? and (2) What is the extent of participation by consumers or HCPs?

### Stage 2: Identifying the Relevant Studies

The search strategy was developed by authors JFT and RAY. Six scientific databases were searched (Web of Science; Scopus; Embase; PubMed, including MEDLINE; CINAHL; and Computer & Applied Science Complete) for research articles published in English between 2010 to 2021. Furthermore, a reference list search (ie, backward reference search) and a cited reference search (ie, forward reference search) were carried out based on the full-text papers that met the study selection criteria. The reference search was repeated on newly identified papers until no additional relevant papers could be found. Multimedia Appendix 1 presents the full search strategy for each database.

### Stage 3: Selecting Studies

The inclusion and exclusion criteria are presented in Textbox 1.

Five authors (JFT, RAY, THD, RM, and NW) independently screened the titles and abstracts of the studies to identify the ones for potential inclusion. Disagreements about exclusions were discussed until a consensus was reached. The full texts of the articles were then independently reviewed by 2 authors (JFT and RAY), who decided whether or not they met the selection criteria. In the case of disagreement, a third author (NW) made the final decision.

**Inclusion and exclusion criteria.**

**Inclusion criteria**

- Adverse drug reactions (ADRs) in the Australian health care system (medication management context)
- Cross-sectional or survey-based studies

**Exclusion criteria**

- Not related to drugs
- Not related to ADR reporting, monitoring, or handling (eg, efficacy or effectiveness of a study’s design)
- Not related to patients’ reporting (eg, a safety study in animals)
- Qualitative studies, review manuscripts, editorials, letters, and news

### Stage 4: Charting the Data

Two reviewers (JFT and RAY) identified characteristic data sets to describe the articles, which included the author(s), year of publication, setting (where the study was conducted, eg, hospital and community pharmacy), study population (eg, physicians), gender, age, intervention, and reporting rates. The data were represented in a logical and descriptive manner in line with the objectives of this review. As per guidelines, scoping reviews do not exclude research based on the research quality but rather identify areas that are lacking in research [23,24]. Therefore, methodological quality or risk of bias of the included articles were not needed [23,24].

### Stage 5: Collating, Summarizing, and Reporting Results

The information extracted were summarized, organized, and discussed. A PRISMA (Preferred Reporting Items for Systematic reviews and Meta-Analyses) flow chart was developed to summarize the identified literature and study selection steps. The narrative and a numerical summary of the characteristics of the included studies were tabulated. In the conceptual analysis, the study aimed to describe 2 main themes: (1) how ADRs are monitored, handled, or reported in the Australian health care system and (2) the extent of participation by stakeholders. The results of the first theme are presented in Multimedia Appendix 2 as the type of reporting or intervention, PV system used, and the focus of the surveillance. The results of the second theme are presented by the study population, setting, and reporting rates.

### Stage 6: Consultation Exercise

The last stage was to engage in a consultation exercise with stakeholders to discuss, inform, and validate the results of the study [23,24]. This scoping review was interdisciplinary [23,24]; the overall expertise covered medicine, clinical pharmacy, PV, epidemiology, public health, health informatics, and statistics. The participation of all the authors throughout the research process served the purpose of constant consultation and provided the rigor and strength to the study findings and conclusions.

## Results

### Overview

**Figure 1:**
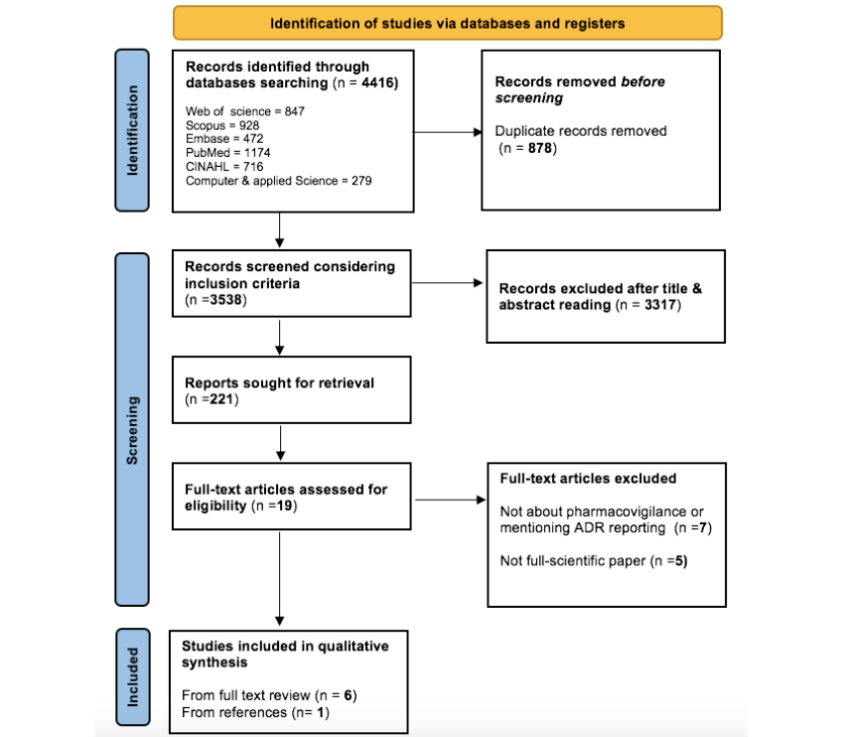
PRISMA (Preferred Reporting Items for Systematic reviews and Meta-Analyses) flowchart of study selection and inclusion criteria. ADR: adverse drug reaction.

Figure 1 (PRISMA flow chart) depicts the full review process and shows the number of citations excluded at each step. A total of 3538 citations were retrieved from the scientific electronic databases after duplicates (n=878) were removed. We screened the titles, abstracts, and full texts using the exclusion criteria and resulted with 6 citations to be included. The reference lists of the selected studies were also manually searched, and 1 article was identified; in total, 7 citations were relevant to the research question and included in the flow chart (stage 3). Multimedia Appendix 3 presents a list of the included studies.

The selected studies were published between 2013 and 2021. Among the studies, 2 focused on consumers and patients, 2 on health care providers, and 3 on information systems. In all, 5 of the 7 studies were in the hospital settings, and the rest were in the community. The findings of the selected studies are presented in 2 themes that relates back to the research questions, “ADR reporting, monitoring, and handling in the Australian health care context” and “participation.” The characteristics of the studies’ findings are summarized in Multimedia Appendix 2.

### ADR Reporting, Monitoring, and Handling in Australia

#### Regulatory Monitoring of ADR

The Australian National Safety and Quality Health Service provides statements for accreditation and include standards for ADR management. Health services are required to have processes for documenting medication allergies and ADRs within the health care records and in the organization-wide incident-reporting system, as well as for submitting major or rare ADRs to the TGA. Providing written ADR information to both the patient and their general practitioner (GP) is a requirement in Australian hospitals [25]. However, despite national and international guidelines for ADR reporting and management, there is no established national protocol for reporting ADRs at the individual health care facility level [26]. As such, there exists substantial interinstitutional variability with respect to the timing and nature of ADR reports [26]. The responsibility for the timely collection and reporting of drug safety information mainly rests with the marketing authorization holder due to mandatory reporting requirements from regulatory agencies [27,28].

#### ADR Handling

An Adverse Drug Reaction Review Committee (ADRRC) is an internal hospital committee comprising of a multidisciplinary team that includes a senior pharmacist and a specialist clinician from at least one of dermatology, immunology, clinical pharmacology, infectious diseases, and general medicine [25,26]. The committee meets every 2 weeks to review all ADR reports; verify diagnoses, if required; organize allergic clinical referrals; and provide further risk mitigation measures through written recommendations to the clinicians involved as well as the patients and carers. Relevant ADR reports are forwarded to the national database at the TGA [26]. In the study of patient satisfaction with an ADR warning card at a tertiary hospital during the period between January 2013 and April 2016 (n=241), patients with suspected ADRs received a wallet-sized paper card titled “Temporary ADR warning card” with the name of the drug thought to be the causative agent. The ADR pharmacist collated the supporting evidence for the case including notes from the inpatient stay and information from the Australian Database of Adverse Event Notifications. The collated evidence was sent to the ADRRC clinicians who reviewed the case and determined an outcome. If the ADR was confirmed, the patient was sent a letter containing the details of the reaction and a laminated card titled “Permanent ADR warning card,” and a copy of the letter was sent to the patient’s GP [25].

#### ADR Reporting Schemes

The Adverse Medicine Events Line is a telephone reporting service that was introduced in Australia in 2003 to allow consumers to report suspected ADRs to the TGA and receive advice about side effects [27]. In June 2014, a pharmacy software vendor, GuildLink, created a web-based Adverse Events Recording module that integrated directly into the TGA ADR web service. This integration allows community pharmacists to report ADRs directly to the TGA from their professional service program instead of having to manually complete a separate ADR reporting form. In 2018, the TGA introduced a black triangle scheme on the packaging of newly approved medicines to alert HCPs and consumers, serving as a prompt to report any ADRs associated with that medicine [28]

### Participants and Extent of Involvement

#### Patient and Consumer

Direct consumer and patient reporting of ADRs to the TGA has been possible since 1964, and since 1990, there had been fewer than 7000 consumer ADR reports [27]. In 2009, there was a spike in consumer ADR reporting (1307/13,298, 9.8%) that was associated to influenza vaccines due to the H1N1 pandemic; however, consumer reporting fell to 3% in 2011 [27]. A study of consumer awareness to ADR reporting between September and October 2012 used 2 methods to survey respondents: a computer-assisted telephone interview (n=2484) and a Pureprofile web-based survey (n=2497). From the 2 groups combined, 46.3% of consumers indicated that side effects of prescription medicines were very common (88.4%) [27]. The awareness of consumer reporting schemes among the whole study group was low (10.4%) [27]. Among 217 respondents who had experienced a side effect and were aware of consumer reporting schemes, 46 (21.2%) reported the ADR, using one of the reporting schemes [27]. Consumers were more likely to report ADRs to their doctors or pharmacists than to the pharmaceutical industry [27,28]. Among the consumer who had experienced a side effect, 84.6% reported the event to an HCP, most often a GP [27]. In the study of patient satisfaction with an ADR warning card at a tertiary hospital during the period between January 2013 and April 2016 (n=241), 85% notified their doctor, 67% notified their family, and 40% notified their community pharmacist [25]. The likelihood of the causative agent being available in community pharmacies determined if participants were more likely to consider it necessary to inform their regular community pharmacist of the new ADR (*P*=.001) [25]. The majority of ADRs experienced by participants fell within 3 drug classes: antibiotics (32%), iron infusions (14%), and contrast media (13%) [25].

#### HCPs’ Participation

In the review and analysis of all ADR reports submitted to the TGA, 28% out of 10,981 reports received in 2003 and about 7% out of the 14,400 reports received in 2011 were from GPs [27]. In 2016, the TGA national PV data also highlighted that hospital and community pharmacists were a major source of ADR reporting, at a much higher rate than doctors (physicians; ie, 16% for pharmacists vs 4% for physicians) [26]. In fact, in their submission for the 2015 TGA review of Australian Medicines and Medical Devices regulations, the Consumers Health Forum argued for mandatory requirements for doctors and pharmacists to report ADRs [28]. However, the Royal Australasian College of Physicians expressed the need for Australian physicians to receive payment for completing ADR reports [27]. In the review of voluntary ADR reports by HCPs to an ADRRC from 2012-2016, of the 555 ADR reports, 471 (84.8%) were reported by hospital pharmacists, 52 (9.4%) by doctors, and 32 (5.7%) by other HCPs. The median time from the date of onset of an ADR to submission of an ADR report (ie, to the TGA) was 3 days [26]. In the study of knowledge and perspectives of ADR reporting by community pharmacists between January to February 2017 (n=263), 35.3% (n=82) of community pharmacists reported at least one ADR to the TGA in the previous 12 months, even though 88.4% (n=205) of the pharmacists encountered an ADR in a patient, and 65.9% (n=153) documented ADRs as part of a clinical intervention at least once a month [28]. The documentation of opioid and penicillin ADR reports in hospital electronic health records showed that nurses and pharmacists were significantly less likely than doctors to omit the reaction description and that pharmacists were significantly more likely to enter the correct classification than doctors (53%, 95% CI 50.52-55.47; *P*<.001) [29,30].

## Discussion

To the best of our knowledge, this is the first scoping review to integrate and synthesize the available published and scientific literature on ADR reporting within the Australian health care context. Thus, this study provides a broad overview of the Australian PV landscape and may highlight potential areas for innovation or quality improvement.

### Consumers Participation in ADR Reporting

The results of our review indicates that consumer reporting is low, as reported in other studies [27]; despite this, consumers who are aware of reporting schemes may be willing to report. The studies showed that consumers were generally more likely to report ADRs to doctors (physicians) or pharmacists [27,28]. The findings are consistent with a recent study reporting that community pharmacists are usually the first point of contact regarding medication-related issues as the most frequently visited HCPs in Australia [10]. Introducing measures to facilitate ADR reporting for both consumer and patients and HCPs can be beneficial. A 2020 systematic review of interventions to improve ADR reporting also concluded that there was scope to include community pharmacies to improve ADR reporting [22]. These findings were also reported in other reviews [20,21]. Robertson and Newby [27] published in 2013 that only 10.14% of consumers (respondents) were aware of ADR reporting schemes. In 2020, Li et al [31] reported the findings of a black triangle scheme (ADR awareness) introduced by the TGA in 2018 to increase awareness and promote the reporting of ADRs by consumers and HCPs [31]. The effect of the black triangle scheme was only marginally successful at increasing ADR reports, that is, there was an improvement in the overall quality of ADR reports submitted but no meaningful increase in the quantity of reports [31]. The study concluded that additional strategies were required to enhance the overall PV system in Australia [28,31]. Considering that there has only been a marginal change in ADR reporting since 2013 [27], it suggests that there is opportunity for regulators to provide more awareness, such as education and further research. The provision of feedback to consumers about their ADR reports and involving consumers in the ADR management process, for example, the provision of ADR Warning cards, resulted in a high level of patient satisfaction and may have positively contributed to patient awareness [25]. In addition, feedback to health care providers on their submitted ADR reports may also encourage further reporting [28].

### Health Care Provider Participation in ADR Reporting

Consumers who experienced a side effect reported the event to an HCP, most often a GP (physician) [27]. However, the review and analysis of all ADR reports submitted to the TGA highlighted a decline in GPs’ contribution, from 28% of ADR reporting in 2003 to 4% in 2016 [26,27]. Therefore, understanding the barriers to and facilitators of ADR reporting among GPs in Australia may inform future interventions. Furthermore, in 2016, the TGA data also highlighted that hospital pharmacists and community pharmacists were a major source of ADR reporting, at a much higher rate than doctors (16% from pharmacists vs 4% from GPs) [26]. A Canadian study exploring why clinicians do not report ADRs posited that previous studies had focused predominantly on the knowledge and attitudes of HCPs and framed underreporting as a failure of individual clinicians without investigating workplace structures or practices that may have influenced reporting [18]. The discrepancies in reporting between GPs and pharmacists are vital and may warrant further investigation into organization and workplace structures. This is particularly important given our results in the documentation of opioid and penicillin ADR reports in a hospital electronic health records that showed that nurses and pharmacists were significantly less likely than doctors to omit the reaction description and that pharmacists were significantly more likely to enter the correct classification than doctors [29,30]. It is important to note that the study did not mention the reasons for reporting differences, leaving room for further investigations. Conversely, despite nurses being less likely to omit the reaction description, their perspective and reporting rate were not discussed and would benefit from further exploration. Nevertheless, although new technologies, for example, “electronic health records,” present as an opportunity to facilitate ADR reporting [21], it has been noted that in practice, these electronic systems can cause unexpected errors, desensitize clinicians to alerts, and increase the documentation burden [19]. This is evident in our findings, where in June 2014, a pharmacy software vendor, GuildLink, created a web-based Adverse Events Recording module that integrated directly into the TGA ADR web service [28]. This allowed community pharmacists to report ADRs directly to the TGA from their professional service program instead of having to manually complete a separate ADR reporting form [28]. In the follow-up from the GuildLink intervention, the quantity of reports received by the TGA was nearly as high as that of the previous year (2013); however, despite the positive start, the numbers declined again in 2015 [28]. The factors that may have influenced a sustained adoption of the GuildLink system were not mentioned or discussed. As new digital technologies emerge and continue to transform health care management [32], exploring existing technology and their shortcomings, for example, GuildLink, and evaluating their health impact present areas of opportunity for improving health quality and patient safety.

Repeated calls for mandated ADR reporting for HCPs have been made, and as discussed above, patients are more likely to discuss or report issues with their medications to the HCPs who initially dispensed or prescribed them (GPs or pharmacists). The Consumers Health Forum in their 2015 submission argued for mandatory requirements for doctors and pharmacists to report ADRs [23,28]. However, the Royal Australasian College of Physicians also expressed the need for Australian physicians to receive payment for completing ADR reports [27]. Furthermore, one of the studies in our results suggested financial incentives for ADR reporting was also highly regarded as a measure to improve reporting by pharmacist [28]. Considering these points, none of the studies in our results explored or noted the impact of mandatory reporting or incentives, for example, financial, on ADR reporting. Furthermore, the finding of the review also showed that despite national and international guidelines for ADR reporting and management, there is no established gold standard systems (policy or guideline) at the individual health care facility level in Australia [26]. Therefore, there exists substantial interinstitutional variability with respect to the timing and nature of reporting ADRs [26]. It was also not possible to pinpoint the net effect of interinstitutional variability in ADR reporting to the TGA, which requires further investigation.

### Strength and Limitation of the Review

The strength of this scoping review is that it is the first review to attempt an integration and synthesis of the available literature on ADR reporting, monitoring, and handling in the Australian health care context; the extent of participation; and ADR reporting systems. Notwithstanding the value of this research, some limitations must be acknowledged. This review was limited to papers published in English; it is possible that other potentially relevant literature in other languages were omitted. Although this review was performed using multiple major databases, searching other databases may have yielded other relevant published papers. A quality assessment of the studies included in the review was not undertaken; however, it was not relevant for the aim and not always necessary for scoping reviews [23,24]. There was limited data from the included studies in regard to the variabilities in participants’ age, gender, or educational status. The majority of the studies were undertaken in the hospital setting and predominately focused on pharmacists and physicians; therefore, applying these results to the entire Australian health care system may require additional considerations, for example, the impact of age, gender, task, or work setting. Different states, regions, and countries may differ in their infrastructures, capacities, general workplace culture, medical education programs, and economic status, which limits the generalizability of the findings of this review.

### Conclusion

The exploratory nature of the scoping review helped to integrate and synthesize the literature to give us a broad overview of the PV and ADR-reporting landscape in Australia. This review is beneficial for identifying research gaps and serves as a vital step to identify key areas for digital interventions and quality improvement in ADR reporting. It is also hoped that the results of this scoping review send a message to regulators (eg, the TGA), software vendors, or policy makers concerning the work that is needed to develop a robust and standardized PV system in Australia. This review did not find a generally accepted “gold standard” protocol or framework for ADR reporting among individual health care facilities, and there is substantial interinstitutional variability with respect to the timing and nature of ADR reporting in Australia.

Despite the marginal effects from previous interventions aiming to enhance ADR reporting, for example, ADR awareness (black triangle scheme) or digital health interventions (GuildLink), there is opportunity to improve patient safety in relation to ADRs. Further evidence-based research is needed to guide ADR interventions design and implementation. To support these interventions, knowledge of the barriers and enablers to ADR reporting among consumers, pharmacists, physicians, and nurses at a primary health care institutional level is warranted.

## Appendix

Multimedia Appendix 1 Full search strategy for each database.

Multimedia Appendix 2 Characteristics of the study findings on adverse drug reactions (ADRs) reporting, monitoring, and handling.

Multimedia Appendix 3 List of studies selected for data analysis and synthesis in this scoping review.

## Abbreviations

ADR: adverse drug reaction
ADRRC: Adverse Drug Reaction Review Committee
GP: general practitioner
HCP: health care professional
PRISMA: Preferred Reporting Items for Systematic reviews and Meta-Analyses
PV: pharmacovigilance
TGA: Therapeutic Goods Administration
