# Supplementary figures and images for: Reporting, Monitoring, and Handling of Adverse Drug Reactions in Australia: Scoping Review
Source: JMIR Public Health Surveill. 2023 Jan 16;9:e40080. doi: 10.2196/40080 (PMC9887513; doi:10.2196/40080)

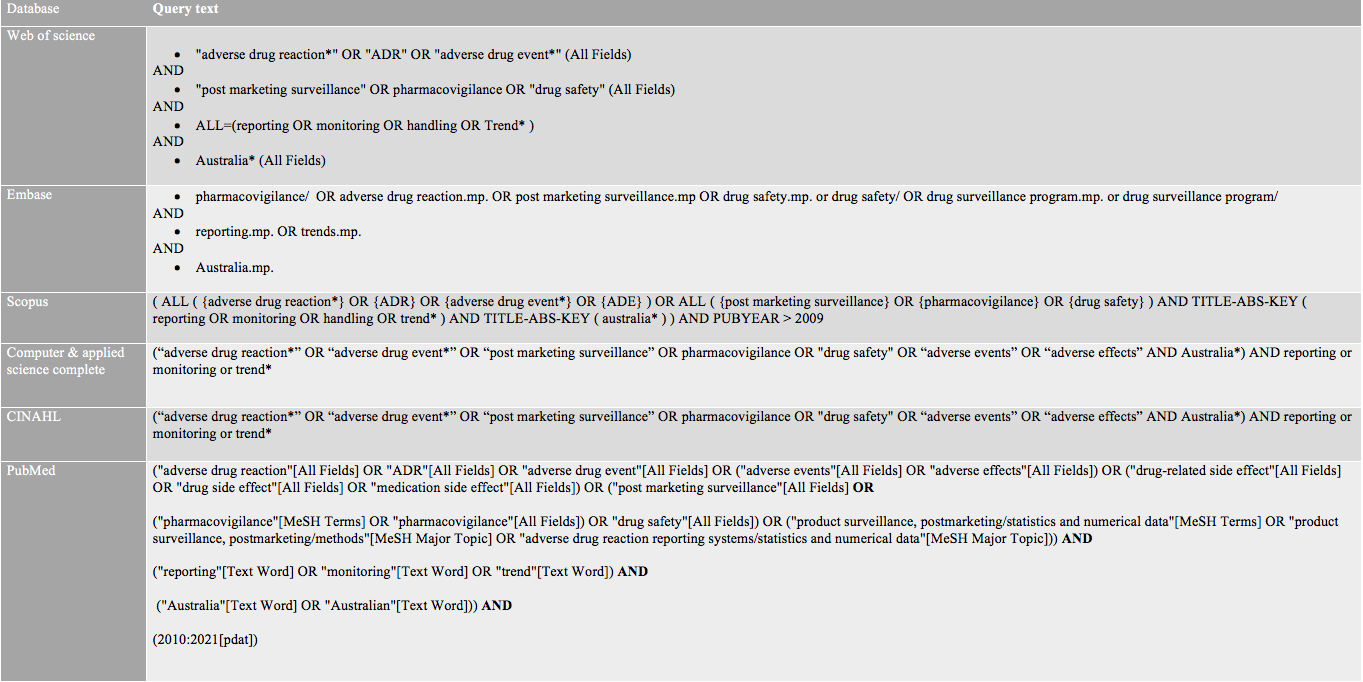

Supplement: Multimedia Appendix 1 [file publichealth_v9i1e40080_app1.png]

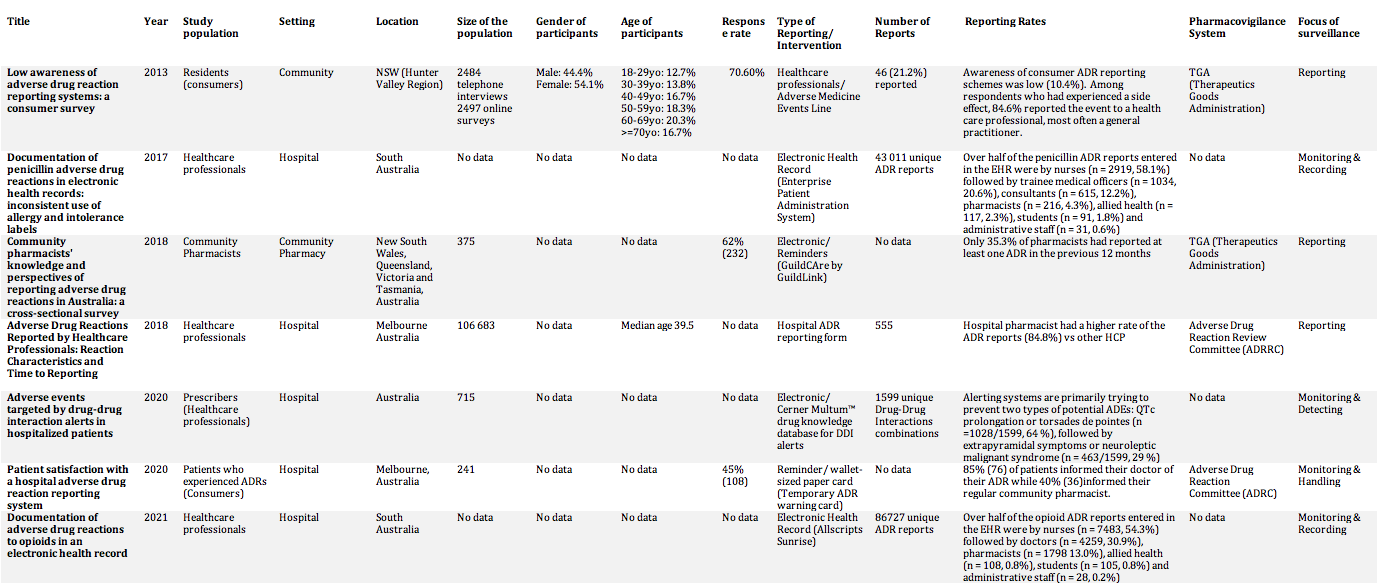

Supplement: Multimedia Appendix 2 [file publichealth_v9i1e40080_app2.png]

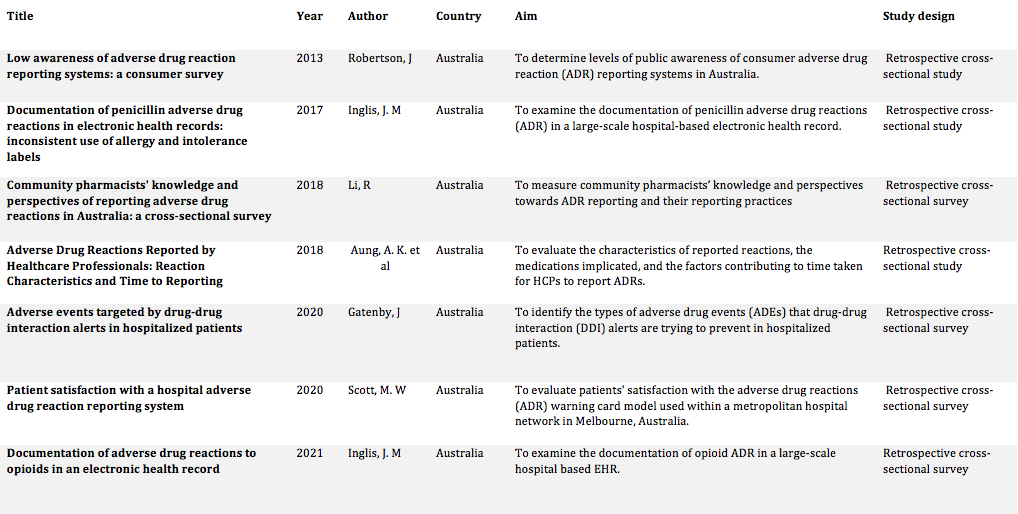

Supplement: Multimedia Appendix 3 [file publichealth_v9i1e40080_app3.png]
